# Supplementary material for: Are carbon emissions trading and green financial instruments synergistic? -Comprehensive quantitative research based on content analysis
Source: PLoS One. 2024 Mar 7;19(3):e0298601. doi: 10.1371/journal.pone.0298601 (PMC10919601; doi:10.1371/journal.pone.0298601)
Supplement: S1 Data — (ZIP) [file pone.0298601.s001.zip › Data/地级市Z_F工作报告.zip!/║╙▒▒/╟╪╗╩╡║╩╨/└┤╫╘╣1⁄2╓┌║┼í╛┬φ┐╦╩2╛▌═°í┐_2012.docx]

各位代表：

现在，我代表市人民政府向大会报告工作，请予审议，并请市政协委员和列席会议的同志提出意见。

一、过去一年工作回顾

刚刚过去的一年，是“十二五”开局之年。面对复杂多变的国际国内形势，我们在省委省政府和市委的坚强领导下，紧紧围绕科学发展主题和加快转变经济发展方式主线，全力以赴稳增长、调结构、控物价、惠民生，较好地完成了市十二届人大四次会议确定的目标任务，改革开放和现代化建设迈出更加坚实的步伐，实现了“十二五”开门红。

——经济持续平稳较快发展。地区生产总值突破千亿，达到1064亿元，比上年增长12 %。全部财政收入168.7亿元，一般预算收入86.6亿元，分别增长20.2%和20.3%。全社会固定资产投资完成615.1 亿元，增长25.6%。实施千万元以上在建项目440个，完成投资480.2亿元，增长24 %。一批重点项目相继竣工投产或开工建设。产业聚集区（园区）步入发展快车道，建成区新增面积达20.57平方公里，完成固定资产投资、规模以上工业增加值分别占全市的52.9 %和80.8%。社会消费品零售总额394.4亿元，增长17.8%。金融机构本外币贷款余额1082.1亿元，比年初增加147.7亿元。

——农业农村发展势头良好。全面落实各项惠农政策，全年投入“三农”资金32亿元，增长56.2%。农业总产值完成253.7亿元，增长5%。粮食生产再获丰收，总产量达89.2万吨，肉、蛋、奶、蔬菜、果品稳定增长。实施千万元以上农业产业化项目61个，产业化经营率达到65%。农村新增土地流转面积11.5万亩，土地流转比例增长5.5个百分点。县域经济不断壮大，四县实现生产总值496.7亿元。农村生产生活条件持续改善。新改建农村公路265.5公里，改造危房3946户，新建沼气池4050个，1万农村贫困人口实现稳定脱贫；除险维修加固水库21座，新增节水灌溉面积14万亩，治理水土流失80平方公里，解决12.8万农村人口饮水或安全问题。新民居建设顺利推进。

——产业结构调整步伐加快。现代服务业主导地位更加突出，初步测算实现增加值285亿元，增长16%；旅游业发展势头强劲，接待国内外游客2127万人次，旅游总收入达172.8亿元，分别增长12.9%和17.3%，荣获全国“最佳休闲城市”称号。港口货物吞吐量2.88亿吨。工业支撑作用更加明显，规模以上工业实现增加值333.2亿元，增长15.6%；装备制造业增加值占规模以上工业的比重达到28.4%。科技对经济增长的贡献率不断提高，实施工业企业技术改造项目256个，完成投资105.8亿元，超出省下达年度计划的32%；高新技术产业增加值占规模以上工业的比重达到25.4%。荣获“2011中国十大最具成长力创新型城市”称号。节能减排力度持续加大，预计单位生产总值能耗下降3.66 %，化学需氧量、氨氮和二氧化硫排放量分别削减1.52%、1.53%和1.5%。

——城乡建设水平进一步提升。以“16+3+2”省定暑期重点项目为引领，城市对标活动为动力，按照5A级景区标准规划全市域，推动城乡面貌再上新台阶。北戴河机场、津秦客专、承秦高速、秦皇岛火车站改造等重大工程按计划推进，昌黄连接线、抚南连接线竣工通车。山海关机场开通11条航线。三个城市区实现出租车通运，客运环境明显改善。高质量完成北戴河火车站及站前广场改扩建、森林湿地公园二期、海上音乐厅、浅水湾浴场等省定暑期重点项目。17条主干道景观整治提升取得显著成效，改造沿街建筑509栋。完成“一场、两带、四线、二十四景”景观亮化工程，新建“北戴河列车”等93组城市景观雕塑。团结里市场改造等工程相继投用，石河生态观光带等一批新景观新亮点建成开放。城市区“六河”治理一期工程完工，县域“七河”深度治理扎实推进。新增绿地面积82.9万平方米、造林面积13.1万亩，森林覆盖率达41.98%。全市新增变电容量72.15万千伏安，新增宽带网用户11.67万户。以县城为重点的小城镇建设步伐加快，全市城镇化率达到49%。

——改革开放实现新突破。沿海地区发展战略启动实施，全国首批服务业和旅游综合改革试点工作全面展开。我市被确定为国家创新型城市试点。成功申列国家公共文化服务体系示范区，经营性文化单位全部完成转企改制，被评为全国文化体制改革先进城市。北戴河新区理顺管理体制和接收工作稳步推进，并被国家住房和城乡建设部确定为全国绿色节能建筑示范区。秦皇岛开发区新兴产业聚集区征地拆迁、基础设施建设全面实施。事业单位绩效工资改革全部完成。政府办基层医疗机构基本药物零差率销售全面实行，公立医院改革试点进展顺利。新耀华集团正式挂牌运营。小额贷款公司达到7家，全年累计贷款6.12亿元，有力支持了“三农”和小微企业发展。民营经济发展活力进一步增强，增加值占全市地区生产总值比重达66.4%。成功举办系列招商活动，一批重大项目签约落地。全年实际到位外资6.02亿美元、内资181.3亿元，分别增长21.1%和66.6%。完成进出口总额43.5亿美元，其中出口22.2亿美元，增长17.9%。

——各项事业全面进步。财政对社会事业支出69.2亿元，增长17.9 %。义务教育办学条件明显改善，中小学校舍安全工程稳步实施，高中教育教学质量不断提高，学前教育三年行动计划全面启动，职业教育持续发展。新建4所县级医院、11所中心乡镇卫生院、342个村卫生室，城市区社区卫生服务实现街道全覆盖。市耀华医院和肿瘤医院成功重组，市妇幼保健院综合病房楼建成使用，市中医院迁建工程进行内装修。乡镇综合文化站、文化信息资源共享等文化惠民工程深入推进，全民健身运动广泛开展。建立山桥集团博士后工作站，全市企业博士后工作站数量达到6家。市档案馆建成投用。食品安全综合抽检合格率和安全食品市场占有率均达94%以上。国防动员和双拥共建工作扎实开展，广大军民在经济建设、国防建设、抢险救灾等方面谱写了风雨同舟、协同奋战的动人篇章，荣膺全国双拥模范城“五连冠”。社会治安和安全生产形势持续稳定好转。第一届北戴河全国科技成果巡回展成功举办，暑期工作任务圆满完成。人口自然增长率为5.26 ‰。审计、统计、妇女儿童、慈善、残疾人、民族宗教、外事侨务、社会公益、气象、人防、地震、科协、地方志等各项事业取得新成绩。

——人民生活持续改善。城镇居民人均可支配收入19385 元、农民人均纯收入7365元，分别增长13.2% 和18.5%。新增城镇就业4.8万人、农村劳动力转移就业4.2 万人。各类社会保险扩面任务顺利完成，在省内率先实现城乡居民社会养老保险制度全覆盖，城镇职工基本医疗保险市级统筹工作走在全省前列。城乡低保标准大幅提升，“五位一体”养老服务体系初步形成。新型农村合作医疗筹资标准提高到每人每年230元，参合率达95.7%。开工建设保障性住房15119 套，超额完成省下达目标任务，3219户低收入家庭搬入经济适用住房和廉租住房。

——政府自身建设不断加强。自觉接受人大法律和工作监督、政协民主监督和社会监督，对矿山整治、水系治理、城市交通、财政风险等重点问题高度重视，全年办理人大代表建议和政协提案550件，办复率100%，满意率达98%以上。改进政务服务工作，整合市长公开电话和政务服务热线，强化网络问政平台建设，民声通道更加通畅、便捷。大幅削减行政审批事项，下放审批权限，开展治理“庸散懒”、优质服务窗口创建等活动，行政效能进一步提升。制定并落实加强法治政府建设的意见，健全制定规范性文件公众参与机制，加大行政执法过错责任追究力度，加强和改进行政复议工作，依法行政能力进一步增强。全面推行行政权力公开透明运行，深化“机制+科技”防治腐败模式，集中开展工程建设领域、公务用车等专项治理，廉政建设和反腐败工作取得新的进展。

各位代表，过去一年，在环境复杂、条件困难的情况下，取得这样的成绩来之不易，这是省委省政府和市委总揽全局、正确领导的结果；是市人大、市政协及社会各界有效监督、鼎力支持的结果；是全市人民和衷共济、团结奋斗的结果。在此，我代表市人民政府向全市人民，向市人大代表、政协委员，向各民主党派、工商联、无党派人士和人民团体，向驻秦人民解放军、武警官兵和政法干警，向中省直机关驻秦各单位，向关心支持秦皇岛发展的海内外朋友们，致以崇高的敬意和衷心的感谢！

我们也清醒地看到，尽管去年多数目标任务完成或超额完成，但受多种因素影响，仍有居民消费价格总水平、社会消费品零售总额、氮氧化物减排3项指标未完成预期目标。经济社会发展仍面临诸多困难和问题：经济实力不够强、发展不够快、质量不够优的问题仍较突出，具有战略支撑作用的大项目、好项目少的问题依然存在；节能减排和产业结构调整优化的任务艰巨；部分企业生产经营困难，经济增长下行压力加大；就业、教育、住房、医疗、食品安全等民生改善工作依然繁重；政府个别部门服务意识不强、效率不高、执行不力的问题还不同程度存在。对这些问题，我们一定高度重视，下大力加以解决。

二、今年工作总体安排

今年是全面贯彻落实省第八次党代会和市第十一次党代会精神的第一年，是实施“十二五”规划承上启下的重要一年，我们将迎来党的十八大胜利召开，做好今年政府工作，意义十分重大。

当前，美欧债务危机仍在持续，世界经济复苏进程充满不确定性，国内经济增长面临的挑战、风险明显增多，但我市发展的有利条件、内在优势和长期向好趋势没有改变。国内经济运行状况总体良好，省第八次党代会开启了建设经济强省、和谐河北的新征程，市第十一次党代会提出了到2015年总量翻番、结构优化、城乡协调、生态一流、社会和谐的发展目标。特别是京津冀区域经济一体化纳入国家“十二五”规划，河北沿海地区发展规划上升为国家战略，秦皇岛被列为国家首批服务业综合改革、旅游综合改革、创新型城市试点、公共文化服务体系示范区和河北统筹城乡一体化发展试点市，这些为我们提供了多载难逢、多重叠加的重大发展机遇。我们将立足当前、着眼长远，进一步强化机遇意识、忧患意识，坚定信心、迎难而上，为率先全面建设小康社会打下坚实的基础。

做好今年的政府工作，要深入贯彻落实科学发展观，紧紧围绕主题主线，以“宜居宜业宜游、富庶文明和谐”滨海名城建设为目标，大力实施旅游立市、产业兴市战略，把稳增长作为首要任务，把调结构作为主攻方向，把改革创新作为强大动力，把统筹城乡作为重要举措，把改善民生作为根本目的，统筹兼顾、积极作为，错位发展、彰显特色，推动经济社会发展更快、更好、更活、更和谐，以优异成绩迎接党的十八大胜利召开。

今年全市经济社会发展的主要预期目标是：生产总值增长10%；全部财政收入增长14%；全社会固定资产投资增长19%；社会消费品零售总额增长17%；出口总值增长10%，实际利用外资增长10%；单位生产总值能耗下降3.66%，化学需氧量、二氧化硫、氨氮、氮氧化物排放量分别削减2.2%、2.1%、3.1%和1.5%；城镇居民人均可支配收入增长10%，农民人均纯收入增长10%以上；城镇登记失业率控制在4%以内；居民消费价格涨幅4%左右；人口自然增长率控制在6.3‰以内。这些目标，综合考虑了经济形势、国家调控和我市实际，既体现了积极进取精神，又注重保持经济增长的稳定性、协调性和可持续性。实际工作中，在保证质量、提高效益、优化结构的前提下，能发展多快就发展多快。

完成今年的各项目标和任务，必须牢牢把握稳中求进工作总基调，以稳增长、控物价、调结构、抓创新、惠民生、促和谐为着力点，努力实现经济社会发展更快、更好、更活、更和谐。更快，就是要加强经济运行调节，努力克服各种不稳定、不确定因素的影响，保持合理投资规模，促进消费增长，稳步扩大出口总值，使经济发展处在一个较快的增长区间。更好，就是要优化产业结构、需求结构、城乡结构，在发展中调整、在调整中提质、在提质中增效，不断放大优势、做靓品牌，增强发展的协调性、特色性和可持续性。更活，就是要始终把改革创新作为提升竞争力、争创新优势的根本动力，以更大的决心和气力解决影响经济健康发展的体制性、结构性矛盾，在一些重点领域和关键环节取得新的突破，不断激发全社会创新创造活力和各类经济主体的发展活力。更和谐，就是要正确处理改革发展稳定关系，加快推进以改善民生为重点的社会建设，加强和创新社会管理，办好一批涉及民生的大事要事，积极有效化解各种矛盾和风险，确保社会和谐稳定。

我们坚信，有市委的坚强领导，有全市人民的齐心协力、奋勇拼搏，一定能够圆满完成今年经济社会发展的各项目标任务，在新起点上开创科学发展、富民强市的新局面。

三、保持经济平稳较快发展

重点做好六方面工作。

（一）推动区域经济快速发展。抢抓河北沿海地区发展规划上升为国家战略契机，统筹投资、消费、出口三者关系，持续加大项目建设、园区发展、招商引资、对外开放工作力度，不断扩总量、增实力、蓄后劲。

一是落实沿海地区发展规划。根据国家和省的要求，制定并落实具体实施方案，找准沿海地区发展战略和京津冀一体化战略的结合点，明确项目重点、责任分工和时序步骤，全面启动新一轮开发建设。进一步明确各县区、开发区、北戴河新区的战略定位、空间布局、重点任务和推进措施，统筹编制完善港口、产业、城市、交通等专项规划，以加快北戴河新区开发建设为引领，着力破解体制机制、港口布局、要素保障、产业聚集等方面的难点问题，力促沿海地区基础设施建设、产业聚集能力、管理水平大幅提升，率先把我市沿渤海、环京津的难得双重优势转化为现实生产力。

二是突出抓好重点项目建设。扩充规模与调优结构并重，细化目标、强化责任，加大工作力度，加强督导调度，提高投资强度，加快建设进度，促进经济总量提升、产业结构优化。全年安排千万元以上项目400个以上，完成投资570亿元以上，比上年增长20%。其中，投资超亿元项目170个，完成投资450亿元，增长18.4%；先进制造及高新技术类项目80个，完成投资200亿元，增长17.6%，确保康泰医学医疗器械技改扩建等100个项目竣工投产。努力破解项目建设瓶颈，在加大向上争取力度的同时，向空中、地下、滩涂挖潜要地，积极缓解用地矛盾；加强投融资平台建设，打破现有条块管理格局，整合优良资产，打造2－3个集投资主体、融资载体、经营实体等于一体的大型集团公司；搞好政、银、企对接，千方百计保证投资进度。围绕“一中心三基地”产业定位，严格准入门槛，充实、优化重点项目库，着力提高战略新兴产业类、文化旅游类、重大基础设施类、保障改善民生类项目比重，严控高耗能行业增长，争取更多项目纳入国家和省建设重点。

三是切实加快产业集聚步伐。狠抓园区基础设施建设，年内完成投资50亿元以上，90%的园区实现“九通一平”，力争储备土地5000亩，争取抚宁骊城、山海关临港开发区晋升省级开发区，实现各县区都有省级园区。努力引入投资强度大、科技含量高、产业关联度高的项目，增强产业集聚效应，培育经济增长极。北戴河新区重点推进国际旅游度假中心、葡萄岛综合旅游、圣蓝皇家海洋公园等已开工项目建设，力促一批新项目开工建设；借鉴先进理念，以选定区域内单体建筑为突破口，加快建设国家绿色节能建筑示范区，推动绿色、低碳、可持续发展。秦皇岛开发区重点加大老区改造升级力度，加快新区开发建设，完善“一区多园”模式，切实在全市整体实力提升、产业结构调整和城乡统筹发展上发挥领跑作用。进一步理顺葡萄酒产业聚集区、西部工业区、大北部工业区、青龙经济开发区管理体制，成立或引进开发建设投资公司，争取在配套设施建设和项目引进上取得新突破。抓紧完善东部临港、北戴河机场空港物流园区规划，引进现代物流业龙头企业和项目，加快形象进度。同时，积极做好园区拓展、新园区谋划工作。

四是不断提高对内对外开放质量和水平。进一步强化开放意识，实行更加积极的开放战略，不断拓展开放领域和空间。突出产业招商和园区招商，深入研究国内外产业转移趋向，以及大公司大集团特别是央企的发展动向，选择主攻重点，明确牵头领导和负责单位，强化常态化管理，积极展开对接。推进以商招商，抓好落户企业后续服务，鼓励其增资扩股、技术改造并借力开展招商活动。坚持集中与分散相结合，开展好灵活多样的招商活动。选择有基础的重点地区举办秦皇岛招商周活动，在日韩、香港、京津唐等地开展小团组招商活动；建立健全园区项目信息库，统筹园区招商活动，做到资源共享、错位发展。积极改善出口产品结构，加快昌黎水产品、戴卡轮毂、康泰医学等出口基地发展，支持优势企业建立域外资源能源基地，开展对外承包工程和劳务合作，着力提升外经外贸规模和水平。

五是有效扩大消费需求。积极挖掘消费潜力，提高企业职工最低工资标准和退休人员基本养老金水平，落实事业单位绩效工资制度，扩大工资集体协商覆盖面，逐步建立完善职工工资支付保障机制；优化消费市场布局，加快深圳茂业等中心商务区建设，力促万达、家乐福等商贸项目落地；建立健全农副产品流通体系，抓好城区农贸市场、农村集贸市场、家政服务网、早餐达标店建设，提升夜经济发展水平；深入推进万村千乡市场工程，保障商品正常供应，确保消费需求不断扩大。加强房地产市场监测分析，完善房地产市场管理，合理引导市场需求，稳定市场预期，确保房地产企业和房地产市场稳健运行。

六是全力抓好工业经济运行。高度重视实体经济的发展，持续加强现有企业的服务保障工作。强化工业运行监控，健全重点监控服务企业数据平台和调度系统，增强调控预警性、主动性。完善工业经济发展联席会议制度和生产要素保障联动机制，认真协调解决企业发展中的困难和问题，严格落实重点企业分级帮扶责任制，持续加强分类指导，确保规模以上工业增加值增长13%以上。

（二）力促旅游、文化、生态互融并进。坚持特色化、差异化发展，以旅游为中心、文化为灵魂、生态为保障，努力构建“旅游+文化+生态”融合发展大格局，不断巩固放大比较优势。

一是进一步加快旅游产业发展。强化大旅游观念和综合产业意识，按5A级景区标准规划控制全市域。以旅游立市百项工程为载体，加快推进栖云山度假区等中高端项目建设，加大集发、老龙头、南戴河娱乐中心等既有景区改造提升力度，确保暑期前有一批新看点。有重点地选择北戴河疗养院所，培育商务会展、培训教育等业态，缓解旅游淡旺季矛盾。加快发展乡村旅游业，开展全国休闲农业和乡村旅游星级示范创建行动，提高望峪山庄、葡萄沟、鲍子沟等景区水平，培育一批山区综合开发建设示范区，打造一批以“绿色、休闲、参与、体验”为主题的优质景区品牌。推进旅游配套设施精细化，建设旅游综合信息服务平台，增设旅游咨询服务中心5处；评定星级酒店6家、乡村星级酒店40家，改造提升家庭旅馆100家，培育一批特色美食、购物娱乐街区。加强旅游市场营销，推行城市休闲国家标准，狠抓旅游市场整治，不断巩固拓展客源市场。力争年接待游客达到2300万人次以上，旅游总收入突破200亿元。

二是进一步推进文化强市建设。抓住文化大发展大繁荣的历史性机遇，着力提升文化软实力。启动以市博物馆为主体的文化艺术中心建设，加快北戴河秦行宫遗址博物馆建设，配合做好河北艺术学院整体迁入北戴河新区工作，新改建县级图书馆2个、县级文化馆4个、乡级文化站26个、村级文化室841个，确保按期完成公共文化服务体系示范区创建任务。加强文化品牌建设，深入挖掘孤竹、碣石、长城等文化内涵，塑造秦皇岛读书节、“七夕”中华爱情节等节庆品牌，谋划推出富有秦皇岛人文特色的大型室内剧目；围绕“确保安全、确保成功、确保精彩、争创一流”的总体要求，办好世界女子拳击锦标赛等赛事。加快休闲体育产业发展，塑造体育名城、健康之城品牌。深入实施文化惠民工程，持续开展“文化广场暑期演出季”等多种形式的群众文化活动，弘扬社会主义核心价值观。促进文化与各领域融合，加快北戴河创意产业园、山海关古城文化产业园、动漫游戏产业基地等项目建设，加强与北京歌华等知名文化公司合作，提升文化产业规模化、集约化、专业化水平。深化文化体制改革，鼓励社会资本投资文化产业，加快培育主导产业、龙头企业。

三是进一步提升生态环境质量。加大生态建设、环境保护力度，争创国家生态园林城市和国家环保模范城市，努力打造全国生态文明先行区。坚持不懈地抓好造林绿化工作，年内完成人工造林8.8万亩、封山育林25.4万亩，新增园林绿地150万平方米。把北戴河近岸海域污染防治作为生态环境建设重中之重，全面实施海洋污染治理、滨海生态修复、水生生态修复与治理等十大工程，统筹兼顾，突出重点，深入推进城区六河、县域七河治理，对入海河流逐个排查整治污染源，建立管理台帐，坚持工程措施和管理措施并举，强化责任落实，坚决打击偷排偷放行为，确保河流、海水水质得到不断改善。抓好港口煤尘、水泥粉尘、工业烟尘等治理，进一步改善空气质量。持续深入地开展“双打”专项行动，强化动态监管，综合运用法律、经济等手段，严惩各种非法占地和非法开采矿产资源行为，全力规范土地管理和矿产资源开发秩序。

（三）推进产业结构优化升级。坚持高端化、新型化、集群化发展方向，深入开展行业对标行动，加快工业化与信息化深度融合，促进传统产业、支撑产业、新兴产业、现代服务业融合协调发展。

一是以更大的力度改造提升传统优势产业。设立市级工业企业技术改造专项资金，理顺工业企业技术改造项目核准备案机制，以贷款贴息方式，支持企业更多引进、利用先进适用技术，不断加大技改投入力度，年内实施技改项目100个以上，促进传统产业提高加工精度、提升产业层次，实现产业链拓展和价值链提升。积极推进抚宁、昌黎、卢龙三县钢铁企业兼并重组和青龙矿产资源整合，引导金属压延业向装备制造业对接，向规模化、高端化、集中化发展。加快耀华集团在线镀膜玻璃、液晶显示玻璃基板等项目建设进度，推进玻璃行业兼并重组。以金海粮油、鹏泰面粉、华夏葡萄酿酒、骊骅淀粉等企业为重点，支持开展技术改造和产品创新，突出深加工、系列化开发，放大粮油食品业竞争优势。

二是以更实的措施做大做强装备制造业。坚持发展整机、壮大配套、培育龙头、推进集聚的原则，突出船舶修造、电力装备、工程机械、高速铁路设备、专用设备等领域，每个县区选择1－2个重点行业，每个行业选择1－2家龙头骨干企业进行重点扶持，以重点行业、骨干企业的率先突破，带动装备制造业新产品研发能力、核心部件制造能力和配套产业带动能力进一步提升。年内重点确保山船重工大型船舶改装修理及海洋工程装备扩建、哈电重装百万千瓦核电辅机产品制造等项目建成投产，山海关船舶配套产业园、戴卡汽车配件产业园、光宇汽车等项目取得突破性进展。

三是以更优的政策培育发展战略性新兴产业。坚持突出重点、完善政策、分类培育。对于数据产业、电子信息、新能源、生物医药等有比较优势的领域，支持其尽快扩规模、提档次。加快秦皇岛数据产业基地建设进度，重点启用数谷大厦，推动物联网技术中心、IBM软件平台等项目尽快投入使用，中兴云计算、交大物联网等项目顺利落地，数据产业研发中心、北大科技产业园等项目取得突破进展。对于高端装备制造、海洋经济等前景广阔的产业，重点是深入调查研究，明确发展重点，制定发展路径和扶持措施，着力引进战略投资者和配套项目。

四是以更宽的视野着力拓展现代服务业。优先发展生产性服务业。积极推进港口总体规划审批工作，实施西港区搬迁改造工程，支持河北港口集团在我市加快发展。制定激励性政策措施，推动煤炭交易市场建设。完善物流网络规划，推进临港物流园区、空港物流园区、龙家营物流园区和抚宁县物流中心建设。谋划建设国际航运服务中心，促进商检、海关等物流服务功能一体化运作，推动港口物流业转型升级。重视发展金融产业，谋划建设秦皇岛金融区，着力引进域外金融机构、金融后台服务中心入驻我市；积极推进新型农村金融机构建设、小额贷款公司试点、金融产品创新；制定政策，积极鼓励和支持各类企业上市融资，努力使金融保险业在增主体、扩规模上有新进展，力争全年金融机构本外币贷款新增160亿元。大力发展生活性服务业，加强社区服务设施改造建设，优先开展老年人居家养老、病人看护、家庭保洁等家政服务。积极培育新兴服务业，整合楼宇资源、调整优化布局，制定针对性政策措施，促进以服务外包、研发设计等为主体的楼宇经济尽快形成规模。加快发展高端服务业，积极推进会议会展中心和北戴河总部大厦建设，加强与京津等周边大城市对接合作，做亮会议会展、总部经济品牌。

坚持把节能减排作为促进科学发展的硬任务，加快产业结构优化升级和转变发展方式的重要抓手，进一步提升工作摆位，加大工作力度。强化重点领域及重点用能企业动态管理，对规模以上工业企业实行月监控，对超过单位产品能耗限额标准用能的，限期整改；情节严重、逾期不治理或者没有达到治理要求的，责令停业整顿或关闭。积极开展能源审计、能耗诊断、能效对标等活动，争取30个重点节能项目提前竣工使用。持续加大淘汰落后产能力度，确保全面完成淘汰落后产能任务。突出减排工作重点，推进现有污水处理厂升级改造，强化城镇污水、垃圾处理设施运行管理，实施电力、钢铁、造纸等行业脱硫脱硝治理工程。抓好抚宁县、秦皇岛开发区及北戴河集发循环经济示范园建设，积极推行清洁生产。加大督导调度频次和密度，推行周调度、月通报、季考核制度和黄牌警告、惩罚性电价、经济处罚等刚性措施；着力健全完善长效机制，从年初到年末由始至终保持攻坚状态，促进节能减排工作常态化、规范化。

（四）统筹城乡一体化发展。以开展全省统筹城乡发展试点为契机，加快农业工业化、现代化和农村城镇化，促进农业增产、农民增收、农村发展，不断开创社会主义新农村建设的新局面。

一是抓好全省统筹城乡一体化发展试点工作。充分发挥全省统筹城乡发展试点市优势，进一步健全统筹城乡发展组织领导和工作机构，充实人员、完善职能，建立目标责任和督导落实制度，形成上下贯通、高效有序的领导体制和工作机制。向上加强沟通对接，向外学习借鉴先进经验，出台统筹城乡试点规划和具体实施方案，明确具体步骤和重点区域，积极探索新模式、新举措。加大在城乡规划、城乡就业、城乡社保、交通建设、水资源管理等重点领域的城乡统筹和资源整合改革力度，确保试点工作取得阶段性成果。

二是增强农业综合生产能力。不折不扣地落实“一产抓特色”战略和各项强农惠农富农政策，强化农业科技创新，全力培育都市型、生态型沿海现代特色农业。全面开展粮食丰产行动，稳定面积、提高单产、增加总产、提升品质。大力发展有机蔬菜，新增设施蔬菜3万亩。加强畜牧业科技支撑、疫病防控等体系建设，创建10个以上部省级标准化规模养殖示范场。肉、蛋、奶、果品、水产品产量分别达到33.8万吨、12.7万吨、10.5万吨、75万吨和40万吨左右。认真落实基层公益性农技推广服务体系建设“一衔接两覆盖”工作要求，深入实施优种工程，加快先进适用技术推广应用。强化农产品质量安全全程监管，新增无公害农产品60个，绿色食品、有机农产品各2个，无公害畜产品产地认证企业20家。

三是创新农业经营方式。培育壮大新型农业经营主体，提高农业集约化水平和组织化程度。大力发展农业产业化，加快培育肉鸡、生猪、海洋水产等9大优势特色产业带，启动20个农业产业示范区建设，农业产业化经营率达到66%。发展壮大龙头企业，分别培育年销售额超50亿元的龙头企业1家、30亿元的1家、10亿元的5家。实施品牌战略，省级以上农产品及加工制品优质品牌达25个。积极发展农村各类专业合作组织，新发展农业专业合作社100个。全面加强与省供销总社的战略合作，力争县城商贸综合体项目取得实质进展。

四是改善农村生产生活条件。统筹城乡基础设施和公共服务，推进公共财政向农村倾斜，基础设施向农村延伸，公共服务向农村覆盖。加快秦抚快速路东段建设，西部快速路5月底竣工，承秦高速年底通车；实施国省干线大中修工程156公里，推进县乡公路改造和养护，改造农村公路95公里，健全城乡一体化客运网络，拉近城乡空间距离，打造市域半小时交通圈、20分钟城乡互动生活圈。加强农田水利建设，维修加固堤防30公里、水库36座，新建水池水窖5000座，发展节水灌溉面积3万亩，解决9万人饮水或安全问题。积极开展农村清洁工程，抓好农村“六乱”治理，新建沼气池2000个。稳步推进农村新民居建设，重点建设好100个省级示范村。推进农村实用人才培养工程，建立健全“三农”综合信息服务平台。逐步解决农民工在城镇就业、居住、医疗、子女入学等方面遇到的困难，有序引导符合条件的农民工进城落户。

五是加快发展县域经济。落实全省县域“腾飞计划”，促进县域加快发展。依托产业园区，围绕承接市区主导产业转移或提供产业配套，培育壮大具有本地产业基础和资源条件的特色主导产业，加快形成一批有竞争力的特色产业集群，带动县域经济规模迅速扩张、实力大幅提升，力争四县占全市地区生产总值的比重提高1个百分点以上。推进抚宁、昌黎县城与市区同城化发展、同城化管理，提高县城规划管理水平。加快重点镇扩容升级，促进产业向城镇集聚、人口向城镇集中，培育一批各具特色的产业重镇、商贸强镇、旅游名镇。大力发展劳务经济，转移农村劳动力3.5万人，劳务经济总量达到48亿元，切实增加农民工资性收入。

（五）加快新型城镇化进程。坚持建设与管理并重、形象与利民共举、城镇与农村一体，努力在城镇承载能力、产业聚集能力、城市形象魅力上实现新跨越。

一是着力健全规划体系。全面实施全域发展战略规划，高起点做好北戴河新区总体规划修编和西港片区改造规划、市政基础设施建设规划、中心城区公共服务设施规划等规划的编制研究工作；加强城市总体规划、土地利用规划、产业发展规划的衔接，抓好城市空间布局优化与产业结构调整的有机结合，提升城市发展的产业支撑能力；建立集中统一的规划管理体制，积极推进数字规划建设，打造智慧城市；加强规划执行跟踪管理和执法监察，增强规划权威性、稳定性和连续性。

二是着力完善基础设施。加快北戴河机场建设，力争年底前达到试运营条件。抓好秦皇岛火车站站前广场及配套交通路网体系建设，争取与车站站房同步投入使用。推进与津秦客专同步实施的上跨和下穿路桥建设，加快龙港路等快速路建设进度，完成先锋路一号桥、北环路汤河桥、经文路和背街小巷等改造工程，打通断头路，疏通微循环。实施北戴河新区等重点污水处理厂建设，抓好市中水回用及北戴河西部水厂、集中供热、北戴河新区智能电网等工程实施，加快市第二垃圾焚烧发电厂建设，进一步完善公共服务配套设施。

三是着力塑造特色魅力。加快金梦海湾、海阳镇、海港区西部四村等重点片区综合开发进度，积极稳妥推进秦皇岛火车站及周边片区、海港区金三角、山海关兴华市场等片区的改造开发。改造和提升海港区西浴场，完善滨海栈道照明、指示等功能。积极推进公园、游园建设，抓好森林湿地公园提升、绿廊绿道建设、小街小巷和老旧小区绿化改造等园林绿化重点工程，加强县城园林绿化，努力在绿量增加、树种优化、立体绿化方面实现新突破，不断增加市民生态、公益活动空间。持续抓好街区改造亮化和街景整治工作，推动昌黎行政中心区域建设、抚宁南戴河改造提升、卢龙古城开发、青龙新城建设，推出一批高品位城市雕塑，努力在暑期前城市形象魅力有一个新跃升。

四是着力提升管理服务水平。坚持科学化、规范化、精细化，以市区环卫体制改革试点为突破口，政府主导与市场化运作相结合，推进管理理念和方式创新，逐步构建科学高效的管理体制和运行机制。实施数字城管升级工程，完善市级数字城管指挥系统，实现数字城管向各县和秦皇岛开发区延伸覆盖，加快城市管理的科技化进程。围绕群众关心的老旧小区物业管理、建筑垃圾运输、环卫保洁等重点领域，加大治理力度，提高城市管理层次和群众满意度。研究制定并启动实施城市综合交通规划，切实改善交通环境。

（六）加大改革创新力度。坚持深化改革，着力加强自主创新能力，通过改革引领、创新驱动，不断激发潜在活力、提高核心竞争力。

一是深化重点领域和关键环节改革。事业单位分类改革，完成全面清理规范工作。财税体制改革，严格财政预算和收支管理，全面推行绩效预算和绩效评价工作；适应国家信贷政策调整变化，积极争取各级支持，合理安排建设项目和实施进度。完善政府性债务管理机制，妥善防范财政风险；统筹使用各类专项资金，建立以贷贴息为主的财政支持发展方式，放大财政资金的引导作用和规模效益；加大财税征管力度，建立综合治税协调机制，促进财税收入持续快速增长。国有企业改革，加快奥莱特腈纶公司以破产重整为主要方式的改制步伐，完成新港港务公司股权、债权及相关资产回购和中阿化肥公司一、二期资产整合。稳步推进经营性国有资产集中统一监管，做大做强国有资本总量，提高运营质量。农村综合配套改革，探索农村产权制度改革，积极稳妥推进农村土地承包经营权流转，土地流转面积增长2个百分点以上。完善村级集体财富积累机制，加快推进基层供销社体制改革。做好工商、质监行政管理体制调整相关工作。

二是做好全国首批旅游和服务业综合改革试点工作。进一步整合两个试点领导小组及办公室职能，突出北戴河和北戴河新区重点，持续加大督导调度和指导服务力度，坚定有序地落实好各项目标要求和政策举措，确保改革试点工作取得实质性成果。加大向上争取力度，切实将试点优势转化为具体政策支持和发展推动力。北戴河新区和北戴河区结合自身特点，选准切入点和突破口，明确实施路径，先行先试，大胆创新，尽快在管理体制、工作机制、发展模式等方面取得新突破，切实把北戴河建设成率先领跑的典范，把北戴河新区打造成示范带动的标杆。

三是推进以科技创新为重点的创新型城市建设。着力建设创新平台，培育创新型单位20个，新建技术研发机构5家，争取天业通联重型装备工程技术研究中心纳入省级序列，专利申请量增长18%。积极促进产学研合作，鼓励开展联合技术攻关，推进隧道通用架桥机组、三维互联网技术等重大科技项目。加快秦皇岛开发区3平方公里科技成果转化示范区建设，积极引进高端合作项目，辐射带动秦皇岛开发区整体成为高新技术成果转化基地。强化科技服务，建立健全市县两级生产力促进中心，加快推进国家科技孵化器二期工程和科技信息服务平台以及北戴河国家科技展览中心建设。深入开展科技特派员基层创业行动。深入落实中长期人才发展规划纲要，以“人才特区”建设为突破口，创新完善人才引进、培养、使用政策体系，全面加强人才队伍建设。

四是支持民营经济加快发展。全面落实加快民营经济发展各项优惠政策，加大财政、税收、土地、金融等方面的扶持力度，帮助解决民营企业特别是小型和微型企业发展面临的困难。继续实施中小企业成长工程，加快培育和发展带动性强、辐射面广的骨干民营企业，年内20家企业进入规模以上企业行列。大力实施品牌战略，提高民营企业自主创新能力，增强内生动力。建立多元化金融服务体系和融资平台，积极开展多种形式的银保企对接合作，加快小额贷款公司建设，为“三农”和小微企业提供融资支持10亿元以上。建立全市中小企业融资服务信息系统，实现银行与企业信息互通。力争民营经济增加值增长15%，占全市地区生产总值的比重达到70%以上。

四、促进社会和谐稳定

高度重视保障改善民生、加强社会建设和社会管理，将工作重点进一步向民生倾斜,财力物力进一步向民生投放,让全市人民得到更多实惠，生活更加幸福。

（一）千方百计扩大就业。强化政府投资、重大项目建设与增加就业联动机制，积极扶持劳动密集型企业增加就业，并向主城区周边和县域转移，实现城镇新增就业4.8万人。加快城乡公共就业服务平台建设，将就业实名制由农村向城镇推行。充分发挥就业专项资金和小额贷款担保基金作用，加大对高校毕业生、就业困难人员和弱势群体帮扶力度。积极推进创业型城市建设，完善市、县区两级创业指导服务中心，新增创业辅导基地2家，力争创业带动就业1万人以上。

（二）完善社会保障体系。加快推进各类社会保险扩面，新农保参保缴费率超过90%以上，实现社会保障卡参保人员全覆盖。建立完善各项社会保障待遇调整机制，努力实现保障水平与经济发展速度、居民收入增长水平、居民价格消费水平相适应。新农合筹资标准提高到290元，参合率保持在95%以上。健全社会救助体系，进一步提高城乡低保标准，城市达到每人每月340－380元，农村达到每人每年2000元。实施扶贫开发“4261”工程，发展扶贫产业项目400个，建设扶贫示范工程200个，实现贫困地区农民人均增收600元，确保1000户稳定脱贫。加大对流浪人员、孤儿等的救助力度。新建街道老年日间照料服务中心5所、社区老年日间照料服务站20所。加强保障性安居工程建设，完成建设任务10600套。

（三）提高教育发展水平。坚持教育优先发展战略，加大教育投入。加快发展学前教育，全面提高普及程度和水平。推进义务教育均衡发展，实施校舍安全工程、薄弱学校改造工程和中小学标准化建设，年内新改扩建中小学60所，逐步消除中小学危房。优化普通高中学校布局，提升整体教育教学质量。做大做强职业教育品牌，提高服务经济社会发展的能力。重视特殊教育工作。支持高等教育发展。提升教师队伍素质。抓好校车安全管理。

（四）积极发展医疗卫生事业。加快医药卫生体制改革，调整医疗卫生资源布局。推进市第二医院整体迁建和市第四医院、军工医院、第三医院传染病救治中心、市120指挥中心建设。抓好青龙满族自治县医院公立医院改革试点工作。新建社区卫生服务中心8个。实现每个行政村有1所标准化卫生室。完成基层医疗卫生机构70%的债务清理化解任务。推进国家基本药物制度向二级以上医疗机构延伸。加强全科医生培养。实施全民健身计划，在100个行政村安装健身器材100套。做好人口计生工作，稳定低生育水平，提高出生人口素质。

（五）维护社会和谐稳定。坚持以人为本、服务优先，创新社会管理，协调推进城乡社区建设，提高城乡社区自治和服务功能。加强信访和调解工作，畅通群众利益诉求表达渠道，认真排查化解社会矛盾纠纷。加强社会治安综合治理，打造“平安秦皇岛”。抓好经济建设秩序的规范治理，坚决惩处在重大工程建设和开发项目中设置障碍、百般刁难、敲竹杠等恶劣行为，严厉打击公然阻挠重点工程建设的邪恶势力。大力开展食品安全专项整治行动，进一步巩固提升食品安全水平。加强基本药物监管，努力创建食品药品安全放心城市。加强市场监管，积极稳控物价。严格落实安全生产责任，加强对重点行业、重点区域、重点时段管控力度，坚决杜绝重大以上安全生产事故；积极推进煤矿整合和各行业专项整治，提升全市整体安全生产水平。健全公共安全应急体系，抓好实战演练，提高应急处置能力。进一步强化“大暑期大北戴河”理念，建立健全暑期工作机制，高标准推进暑期重点项目，全力做好安保警卫、社会稳定、服务保障、环境改善和借力暑期等重点工作，全面提升暑期服务质量水平，打造安全暑期、和谐暑期。

以开展加强基层建设年活动为契机，进一步加强基层民主政治建设，切实搞好村“两委”换届工作。进一步做好国防动员、人民防空、民兵预备役工作，深入开展双拥共建活动。支持工会、共青团、妇联、科协等群众组织开展工作。推动司法行政、民族宗教、外事侨务、防震减灾、气象、地方志、档案等各项事业全面进步。积极发展老龄、残疾人和慈善事业。

五、切实加强政府自身建设

全面完成今年经济社会发展目标任务，必须进一步转变政府职能，着力提升推动科学发展的执行力和创造力，努力以“一个好的精神状态、一个好的工作作风、一个好的工作业绩”，建设人民满意的政府。

（一）加强服务型政府建设。时刻牢记全心全意为人民服务宗旨，把人民群众的需要放在首位，坚持深入基层、深入群众，加强调查研究，千方百计为群众办实事、解难事。建立健全市、县、乡三级联动的政务服务体系，实施政务服务扩域增效工程和标准化认证工作，进一步优化审批流程，提升审批效率。规范政府及公务员服务行为，推行服务质量公开承诺制，开展服务窗口亮化工程，着力提升服务质量和服务形象。加大对中央和省重大决策部署落实情况监督检查力度，确保政令畅通。加强电子政务建设，精简会议、文件，改进会风、文风。

（二）加强创新型政府建设。进一步解放思想，锐意创新，勇于破除陈旧观念和惯性思维，坚持把上级要求与工作实际有机结合，创新发展理念、工作思路、方式方法，积极破解改革建设中的新矛盾新问题，努力使各方面工作更富有特色。建立健全有效的激励机制和保障机制，努力形成鼓励创新、支持改革的良好氛围。坚持统筹兼顾，科学摆布各方面工作，推动经济建设、政治建设、文化建设、社会建设、生态建设协调并进、稳步发展。

（三）加强法治政府建设。坚持依法行政，健全科学民主决策机制，规范公众参与、专家咨询、集体讨论的决策程序，健全政府常务会议旁听、政府部门重大决策会审等制度，不断提升政府科学民主高效决策的能力和水平。全面落实人大及其常委会的决议决定，主动接受政协民主监督，广泛听取各民主党派、工商联、无党派人士和社会各界的意见建议，认真接受新闻舆论和人民群众的监督。进一步加强和改进行政复议工作，积极化解行政争议。以山海关区为试点，加强政府绩效管理，落实行政问责，严格责任追究。

（四）加强廉洁政府建设。严格执行廉洁从政各项规定。着力加强政务服务中心、公共资源交易中心、廉政风险防控中心建设，完善“机制+科技”防治腐败模式。进一步推进行政权力公开透明运行，深化政务公开，对土地管理、工程建设、政府采购等实行阳光操作、阳光运行、阳光监督，对公权私用、吃拿卡要等行为予以严肃处理。牢记“两个务必”，大力倡导艰苦奋斗、勤俭节约之风，坚决反对铺张浪费，严格控制“三公”经费支出。严肃查处违法违纪案件，切实纠正损害群众利益的不正之风。

各位代表！我们面临的任务繁重而艰巨，我们肩负的责任重大而光荣。我们一定要紧密团结在以胡锦涛同志为总书记的党中央周围，在省委省政府和市委的正确领导下，坚定必胜信心，勇敢面对挑战，团结奋斗，开拓进取，推动“宜居宜业宜游、富庶文明和谐”滨海名城建设迈出新步伐，以优异的成绩向党的十八大献礼！
